# Bacteriochlorophyll *f*: properties of chlorosomes containing the “forbidden chlorophyll”

**DOI:** 10.3389/fmicb.2012.00298

**Published:** 2012-08-10

**Authors:** Kajetan Vogl, Marcus Tank, Gregory S. Orf, Robert E. Blankenship, Donald A. Bryant

**Affiliations:** ^1^Department of Biochemistry and Molecular Biology, The Pennsylvania State University, University ParkPA, USA; ^2^Departments of Biology and Chemistry, Washington University in St. LouisSt. Louis, MO, USA; ^3^Department of Chemistry and Biochemistry, Montana State UniversityBozeman, MT, USA

**Keywords:** green sulfur bacterium, bacteriochlorophyll, *Chlorobium limnaeum*, chlorosomes, photosynthesis

## Abstract

The chlorosomes of green sulfur bacteria (GSB) are mainly assembled from one of three types of bacteriochlorophylls (BChls), BChls *c*, *d*, and *e*. By analogy to the relationship between BChl *c* and BChl *d* (20-desmethyl-BChl *c*), a fourth type of BChl, BChl *f* (20-desmethyl-BChl *e*), should exist but has not yet been observed in nature. The *bchU* gene (bacteriochlorophyllide C-20 methyltransferase) of the brown-colored green sulfur bacterium *Chlorobaculum limnaeum* was inactivated by conjugative transfer from *Eshcerichia coli* and homologous recombination of a suicide plasmid carrying a portion of the *bchU*. The resulting *bchU* mutant was greenish brown in color and synthesized BChl *f*_F_. The chlorosomes of the *bchU* mutant had similar size and polypeptide composition as those of the wild type (WT), but the Q_y_ absorption band of the BChl *f* aggregates was blue-shifted 16 nm (705 nm *vs*. 721 nm for the WT). Fluorescence spectroscopy showed that energy transfer to the baseplate was much less efficient in chlorosomes containing BChl *f* than in WT chlorosomes containing BChl *e*. When cells were grown at high irradiance with tungsten or fluorescent light, the WT and *bchU* mutant had identical growth rates. However, the WT grew about 40% faster than the *bchU* mutant at low irradiance (10 μmol photons m^−2^ s^-1^). Less efficient energy transfer from BChl *f* aggregates to BChl *a* in the baseplate, the much slower growth of the strain producing BChl *f* relative to the WT, and competition from other phototrophs, may explain why BChl *f* is not observed naturally.

## Introduction

Chlorosomes are the defining property of green bacteria and are the light-harvesting structures used for phototrophic growth of these bacteria (Blankenship and Matsuura, 2003; Frigaard and Bryant, 2006; Oostergetel et al., 2010; Bryant et al., 2012). Green bacteria include all known phototrophic members of the eubacterial phylum *Chlorobi*, some members of the *Chloroflexi*, and “*Candidatus Chloracidobacterium thermophilum*,” the only known phototrophic member of the phylum *Acidobacteria* (Bryant et al., 2007, 2012). Green sulfur bacteria (GSB) that are green in color produce chlorosomes containing either bacteriochlorophyll (BChl) *d* or BChl *c* and the carotenoid chlorobactene, but brown-colored GSB produce chlorosomes containing BChl *e* and usually the carotenoid isorenieratene (Chew and Bryant, 2007; Maresca et al., 2008; Liu and Bryant, 2012). A single chlorosome can contain up to ~250,000 BChl *c*, *d*, or *e* molecules (Martinez-Planells et al., 2002; Montaño et al., 2003) which self-assemble into one of several different suprastructures (Ganapathy et al., 2009, 2012; Garcia Costas et al., 2011). A GSB cell contains ~200 chlorosomes, and thus a green bacterial cell contains ~50 million BChl molecules, which together account for ~30% of the cellular carbon (Frigaard and Bryant, 2006). These enormous light-harvesting antennas allow green bacteria to grow at extremely low irradiances at which no other phototrophs can survive. Examples include GSB that grow at a depth of ~110 meters in the Black Sea (Manske et al., 2005; Marschall et al., 2010) and a GSB that was isolated at a depth of ~2200 m on the floor of the Pacific Ocean near a black smoker (Beatty et al., 2005).

The chlorophylls (Chls) found in chlorosomes were once commonly referred to as “*Chlorobium*” Chls, and they differ from other (bacterio)chlorophylls [(B)Chls] in several important ways (Chew and Bryant, 2007; Liu and Bryant, 2012). Firstly, although these molecules are commonly referred to as BChls, they are in fact chlorins and have properties more similar to Chl *a* than to those of bacteriochlorins, such as BChl *a*. Secondly, they carry a hydroxyl group at the chiral C-3^1^ carbon atom, and they lack the methylcarboxyl moiety found in all other types of (B)Chls at C-13^2^. These two properties allow BChl *c*, *d*, and *e* to self-aggregate in a protein-independent manner in the interior of the chlorosome (Ganapathy et al., 2009, 2012). Thirdly, these BChls can be methylated at any or all of three positions, C-8^2^, C-12^1^, and C-20, on the periphery of the tetrapyrrole macrocycle (Maresca et al., 2004; Gomez Maqueo Chew et al., 2007). Methylation on the C-20 methine bridge by the BchU methyltransferase converts bacteriochlorophyllide *d* into bacteriochlorophyllide *c*, and causes a red-shift of about 15 nm in the absorption spectrum of both monomeric and aggregated BChls in chlorosomes (Maresca et al., 2004; Wada et al., 2006). This methylation also affects the supramolecular structures that can form inside the chlorosomes (Ganapathy et al., 2009, 2012).

**Figure 1:**
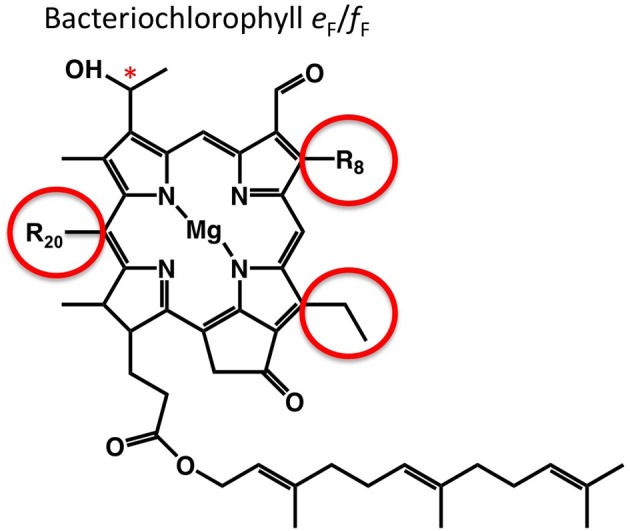
**The structure of BChl *e*_F_ and *f*_F_.** For both BChls, the C-12 side chain is an ethyl group (lower right circle); the R_8_ side chains may be ethyl, propionyl, isobutyl, or neopentyl (upper right circle); and the C-3^1^ carbon may have *R* or *S* stereochemistry (asterisk). For BChl *e*_F_, the R_20_ side chain is a methyl group; for BChl *f*_F_, the R_20_ side chain is a hydrogen.

BChl *e* differs from BChl *c* by the presence of a formyl group rather than a methyl group at the C-7 position of the chlorin ring (Figure 1) (Chew and Bryant, 2007; Liu and Bryant, 2012). An equivalent formyl group occurs in Chl *b*, and in organisms that synthesize Chl *b*, an oxygen-dependent enzyme, chlorophyllide *a* oxygenase, oxidizes the methyl group to produce the formyl group (Tanaka et al., 1998; Tanaka and Tanaka, 2011). This mechanism obviously cannot occur in GSB, which are strict anaerobes, but the alternative enzyme(s) that perform this oxidation have not yet been identified in brown-colored GSB (Liu and Bryant, 2012). In principle, a fourth type of BChl, BChl *f* (20-desmethyl BChl *e*) should occur in GSB, but BChl *f* has to date only been produced by chemical synthesis, and it has never been observed in any natural system (Tamiaki et al., 2011). BChl *f* is analogous to BChl *d* (20-desmethyl BChl *c*) and is identical in structure to BChl *e* except for the absence of the C-20 methyl group (Figure 1). By analogy to the properties of BChl *d*, it would be predicted that BChl *f* aggregates in chlorosomes would have an absorption maximum at ~705 nm (Blankenship, 2004).

The availability of the complete genome sequence for *Chlorobaculum tepidum* (Eisen et al., 2002), and the development of a highly efficient method for natural transformation of this GSB (Frigaard and Bryant, 2001), led to very rapid progress in understanding the photosynthetic apparatus of this model GSB. The ability to construct targeted gene knock-outs and to perform complementation experiments led to the complete elucidation of the biosynthetic pathways for the synthesis of BChl *c* (Chew and Bryant, 2007; Liu and Bryant, 2012), chlorobactene and other carotenoids (Frigaard et al., 2004b; Maresca et al., 2008), chlorosome structure and function (Frigaard et al., 2004a; Li and Bryant, 2009), thiosulfate and sulfide oxidation (Chan et al., 2008, 2009; Azai et al., 2009; Gregersen et al., 2011; Holkenbrink et al., 2011), and other aspects of the physiology and metabolism of this organism (e.g., Tsukatani et al., 2004; Li et al., 2009). However, progress toward understanding the biosynthesis of BChl *e*, and structural and functional properties of the photosynthetic apparatus of brown-colored GSB, has been markedly slower because of the absence of a tractable genetic system in such organisms. In this study we report the construction of a mutation in the *bchU* gene of the brown-colored GSB, *Chlorobaculum limnaeum*, by conjugative transfer of a suicide plasmid from *Escherichia coli*. The resulting mutant produces chlorosomes containing BChl *f*, the BChl that has never been observed in natural GSB populations. The properties of chlorosomes containing BChl *f* are described, and some possible reasons why this pigment apparently does not occur in natural populations of GSB are discussed. These results were presented at the 7th International Conference on Porphyrins and Phthalocyanines, which was held on Jeju Island, South Korea on July 1–6, 2012.

## Materials and methods

### Bacterial strains and growth conditions

*Chlorobaculum limnaeum* strain DSM 1677^T^ (Imhoff, 2003) was obtained from the culture collection of Dr. Johannes Imhoff and was maintained in liquid culture at room temperature in standard SL10 medium for GSB (Overmann and Pfennig, 1989). Although this strain had not previously been reported to grow on thiosulfate, it was found that the strain could grow well on the same medium used for cultivation of *C. tepidum* (Wahlund and Madigan, 1995). *C. limnaeum* cells were acclimated to growth in the presence of 50 μg kanamycin ml^−1^ by gradually increasing the concentration of kanamycin in the growth medium. Cells were grown at room temperature on CL medium (Frigaard and Bryant, 2001) at irradiances of 10–100 μmol photons m^−2^ s^−1^ provided by either tungsten or cool white fluorescent lamps as specified in the text.

Recombinant DNA procedures were performed using chemically competent cells of *Escherichia coli* strain TOP10F′. Conjugation experiments were performed with *E. coli* strain S17-1. *E. coli* cells were grown in Luria–Bertani medium supplemented with 100 μg spectinomycin ml^−1^.

### Conjugative inactivation of the *bchU* gene

The genome sequence of *C. limnaeum* has been determined and will be reported elsewhere (Vogl et al., in preparation). To introduce suicide plasmids into *C. limnaeum* by conjugation from *E. coli*, the mobilizable plasmid pCLCON1 was constructed as follows. The *oriT*-containing region of pLO2 (Lenz et al., 1994) was amplified by PCR by using a proofreading DNA polymerase together with primers pLO2F and pLO2Rev, which included HindIII and EcoRI restriction sites, respectively (Table 1). The resulting PCR product and plasmid pSRA81 (Frigaard et al., 2004b) were digested with HindIII and EcoRI. The resulting HindIII-EcoRI fragment from pSRA81 containing the *aadA* gene, and the digested PCR product from pLO2, were ligated, resulting in plasmid pCLCON. A fragment of the *bchU* gene (GenBank accession JX292262) was amplified by the polymerase chain reaction (PCR) using the following primers: CL_bchUF and CL_bchURev (Table 1), and the resulting amplicon was cloned into the HindIII and PstI sites of plasmid pCLCON to produce pCLCON1. This plasmid was transformed into *E. coli* strain S17-1, in which the genes required for conjugative transfer are integrated into the *E. coli* chromosomes (Simon et al., 1983).

**Table 1:** **Oligonucleotide primers^*a*^**.

**Primer**	**Oligonucleotide sequence (5′ to 3′)**	**Restriction enzyme**
**PRIMER FOR PLASMID pCLCON**		
pLO2F	CTCGAGCAAG**AAGCTT**CCCGTTGAAT	HindIII
pLO2Rev	GGGTTAAAAA**GAATTC**TGCATTAATGA	EcoRI
**PRIMERS FOR CLONING PART OF *bchU* AND VERIFICATION OF CONJUGANTS**		
CL_bchUF	GACAATGAGC**AAGCTT**GACCTCCTGA	HindIII
CL_bchURev	GTAGAGAATG**CTGCAG**AACATCACCG	PstI
bchUtestF	TGACGGCAACCAGCATTGTG	
aadAtestRev	ATCACTGTGTGGCTTCAGGC	

a Primers used to generate plasmid pCLCON and to amplify a part of bchU and to verify the insertion of the plasmid into the genome of C. limnaeum. Sequences in bold indicate the restriction sites introduced for cloning (indicated at the right).

Overnight cultures of the strain harboring pCLCON1 were diluted 1:10 and grown to OD_550 nm_ ~0.6. The cells from a 1.5-ml aliquot of the culture were pelleted and washed three times with CL medium that did not contain sulfide and bicarbonate. The *E. coli* cell pellet was then transferred to an anoxic chamber (Coy Laboratory Products, Grass Lake, MI). *C. limnaeum* was kept on plates because the plating efficiency remained higher than when cells were maintained in liquid media. The mating mixture for conjugation was established as follows. About two inoculation loops of *C. limnaeum* cells were scraped up from a plate and mixed with *E. coli* cells, which were resuspended in 100 μl anoxic CL medium lacking sulfide and bicarbonate. The mixture was spotted onto CPC plates (Wahlund and Madigan, 1995) and plates were incubated in an anoxic jar without a sulfide-generating system at RT until growth was visible. Cell material was then scraped up and streaked onto CP plates supplemented with 30 μg kanamycin ml^−1^, 100 μg streptomycin ml^−1^, and 200 μg spectinomycin ml^−1^. Plates were incubated with a sulfide-generating system until colonies were visible (about 10 days). Several transconjugant colonies were picked and transferred to fresh plates containing the appropriate antibiotics. Single colonies were restreaked three times before further analysis by PCR and high-performance liquid chromatography (HPLC).

### Chlorosome preparation and analysis

Chlorosome isolation was basically performed as previously described (Vassilieva et al., 2002). Cultures of *C. limnaeum* were harvested after 7 days. Cells were centrifuged (7500 × *g*, 20 min) and were resuspended in isolation buffer (10 mM Tris-HCl pH 7.5, 2.0 M NaSCN, 5.0 mM EDTA, 1.0 mM PMSF, 2.0 mM DTT) that additionally contained 3 mg lysozyme ml^−1^; the resulting suspension was incubated at room temperature for 30 min. Afterwards, the cells were mechanically disrupted using a French press at 138 MPa. Chlorosomes were separated from large cell debris and unbroken cells by centrifugation (10,000 × *g* for 20 min). The chlorosomes and membrane vesicles in the supernatant were concentrated by ultracentrifugation at 220,000 × *g* for 2 h. The chlorosomes were separated from membranes on continuous sucrose density gradients (10–53% linear gradients prepared in isolation buffer) by ultracentrifugation at 220,000 × *g* for 18 h at 4°C. The chlorosomes were washed twice with 4 volumes of phosphate buffer (10 mM potassium phosphate pH 7.2, 150 mM NaCl) and pelleted by ultracentrifugation at 220,000 × *g* for 1.5 h. The isolated chlorosomes were resuspended in 1–2 ml of phosphate buffer containing 1.0 mM PMSF and 2.0 mM DTT and stored at 4°C until further required.

Chlorosome proteins were analyzed by polyacrylamide gel electrophoresis in the presence of sodium dodecylsulfate (SDS-PAGE) using a Tris-Tricine buffer system (Schägger and von Jagow, 1987). The stacking gel was 3% monomer and 3.3% crosslinker and the resolving gel was 15% monomer and 3.3% crosslinker. Briefly, samples containing ~20 μg of BChl *c* were incubated at 56°C in 1× loading buffer (0.1 M Tris-HCl buffer, pH 6.8, 24% (v/v) glycerol, 1% (w/v) SDS, 2% (v/v) mercaptoethanol, 0.02% (w/v) Coomassie blue for about 2 min and electrophoresed for 16 h at constant voltage of 70V. Proteins were visualized through silver staining as previously described (Blum et al., 1987).

### Absorption and fluorescence measurements

Room temperature absorbance spectra for whole cells were measured with a GENESYS 10 spectrophotometer (Thermo Fisher Scientific Corp., Waltham, MA), with a Cary 14 spectrometer modified for computerized data acquisition and operation by OLIS, Inc (Bogart, GA), or with a Lambda 950 UV/Vis/NIR spectrophotometer (Perkin Elmer Inc., Waltham, MA). Fluorescence spectra of isolated chlorosomes were measured using a customized PTI fluorometer (Photon Technology International Inc., Birmingham, NJ) consisting of a Xe excitation lamp, excitation monochromator, emission monochromator, signal chopper, lock-in amplifier, and avalanche photodiode detector. For fluorescence emission, the BChl *e* and BChl *f* chlorosomes were excited at their Soret bands, at 457 nm and 446 nm, respectively. Isolated chlorosomes were prepared for spectroscopy by dilution to a Q_y_ band absorption of 0.1 with 20 mM Tris-HCl buffer, pH 8.0. When necessary, the chlorosomes were fully reduced by the addition of sodium dithionite to a final concentration of 25 mM, and subsequent incubation in the dark for 1 h at 4°C prior to measurements. Absorbance and fluorescence spectra of isolated chlorosomes were recorded at 77K by adding glycerol to the samples to a final concentration of 50% (v/v); the sample was then cooled with liquid nitrogen in an Optistat DN2 cryostat (Oxford Instruments, Oxfordshire, UK).

### Dynamic light scattering measurements

Dynamic light scattering (DLS) was used to compare the hydrodynamic diameter (*d*_H_) of the chlorosomes of the WT and the *bchU* mutant. The measurements were performed with a ZetaSizer Nano ZS (Malvern Instruments Inc., UK) in dynamic light scattering mode. All measurements were made in a 1 cm plastic cuvette, at ambient temperature, with back angle scattering detection at 173° to incident beam. The absorbance of each chlorosome sample was adjusted to 0.5 at the Q_y_ maximum using 20 mM Tris-HCl buffer, pH 8.0, before measurements. This technique measures the diffusivity of the particle (D) and calculates *d*_H_ of the particles using Einstein's equation: *d*_H_ = kT/3πηD, where k is the Boltzmann constant, T is the absolute temperature in Kelvin, and η is the viscosity of the solution. Because chlorosomes are not spherical, DLS estimates the diameter of a solvated hypothetical solid sphere having the same diffusion coefficient as the chlorosome.

### Pigment analysis by HPLC and mass spectrometry

Reversed-phase HPLC was used to resolve the peaks of the different homologs of BChl *e* and BChl *f* as previously described (Frigaard et al., 1997). Cells from 1.0 ml of liquid culture were pelleted by centrifugation, and the pigments were extracted by sonication with acetone-methanol (7:2 v/v). The pigment extracts were filtered, and 0.1 volume of 1.0 M ammonium acetate was added to the filtered samples immediately before injection onto the HPLC column. The resulting pigment extract was immediately subjected to analytical HPLC on an Agilent Series 1100 HPLC system (Agilent Technologies, Palo Alto, CA). Pigments were separated on a 25 cm by 4.6 mm Discovery 5 μm C_18_ column (Supelco, Bellefonte, PA) attached to a 1,024-element diode array detector (Model G1315B, 1100 Series, Agilent Technologies, Palo Alto, CA). The resulting data were analyzed using ChemStation software (Agilent Technologies, Palo Alto, CA).

For mass analyses of BChls, pigments were separated by a reversed-phase HPLC system that was directly coupled with a tandem mass spectrometer for MS–MS analysis. The samples were demetallated to form the corresponding pheophytins by post-column introduction of formic acid into the eluent flow as previously described (Airs and Keely, 2000). The resulting data were analyzed with MassLynx software version 3.5 (Micromass, Ltd., Manchester UK). All mass spectrometric measurements were performed at the Mass Spectrometry Facility in The Huck Institutes for the Life Sciences at The Pennsylvania State University (University Park, PA).

### Growth rate measurements

*C. limnaeum* was grown at room temperature (22–25°C) on a rotating wheel that was uniformly illuminated from the front. Two different light sources were used. In some experiments, light was provided by tungsten lamps which are highly enriched in light at wavelengths longer than ~600 nm. In other experiments, light was provided by cool white fluorescent lamps, which are enriched in blue-green light with wavelengths shorter than 600 nm. Before growth rates were determined, cultures were acclimated to the light intensity and the light source to be used for the growth rate measurement. Inocula from the starter cultures were diluted into fresh medium at OD_650 nm_ = 0.05, and the OD_650 nm_ was then monitored until the culture reached early stationary phase. In cultures grown at high irradiance, the transient appearance of polysulfide and elemental sulfur globules interfered with the light scattering measurements for OD_650 nm_ values in the range of 0.3–0.7. Nevertheless, it was still possible to estimate the growth rate from OD_650 nm_ measurements with reasonable accuracy. Under low irradiance conditions, polysulfide and sulfur globules did not interfere with the growth rate determination.

## Results

### Construction and verification of a *bchU* mutant of *C. limnaeum*

**Figure 2:**
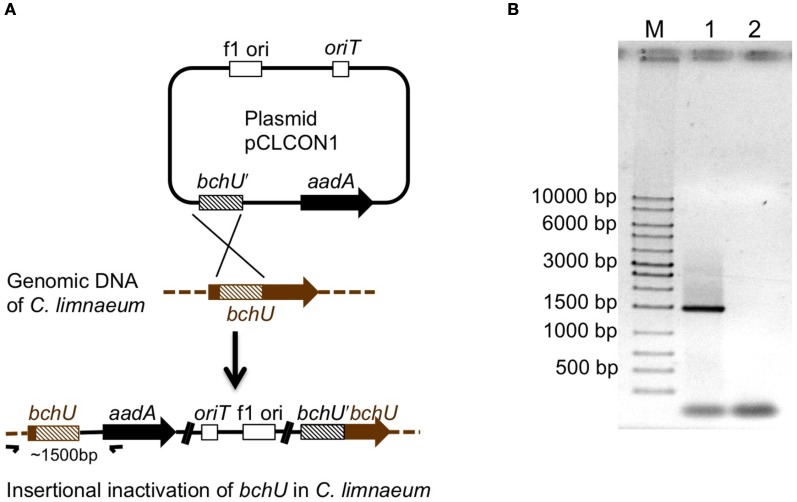
**(A)** Scheme showing the general features and the arrangement in suicide plasmid pCLCON1 and how a single cross-over recombination event between the gene-internal fragment of *bchU* (*bchU′*) leads to insertional inactivation of the chromosomal *bchU* gene. **(B)** Agarose gel electrophoresis of PCR products to demonstrate insertional inactivation of the *bchU* gene. Lanes: M, size markers with sizes in bp indicated; lane 1, the template DNA was from a *bchU* mutant; lane 2, template DNA from the WT strain. The ~1500 bp amplicon resulting from primers flanking *bchU* and the *aadA* (bchUtestF and aadAtestRev; see Table 1) gene (small arrows in panel A) in lane 1 shows that the *bchU* gene has been insertionally inactivated.

As described in the “Materials and Methods,” plasmid pCLCON1 was introduced by conjugation from *E. coli* strain S17-1 into *C. limnaeum*. After selection on plates containing kanamycin, streptomycin, and spectinomycin, brownish green transconjugant colonies arose after about 10 days. Selected colonies were subjected to three rounds of restreaking on selective media, and individual transconjugant colonies were screened by PCR amplification of a fragment spanning the *bchU* gene and the inserted plasmid (see Figures 2A,B). The specific amplification of a ~1500 bp fragment using PCR primers bchUtestF and aadAtestRev (Table 1) from the desired transconjugants (Figure 2B), but not from the WT strain, demonstrated that the *bchU* gene had been insertionally inactivated by a single-crossover, homologous recombination event as illustrated in Figure 2. It was not possible to obtain a PCR product spanning the entire inserted plasmid; however, as will be described below, the phenotype of the resulting mutant showed that no functional copies of the *bchU* gene remained because BChl *e* was no longer detectable in the transconjugant cells.

**Figure 3:**
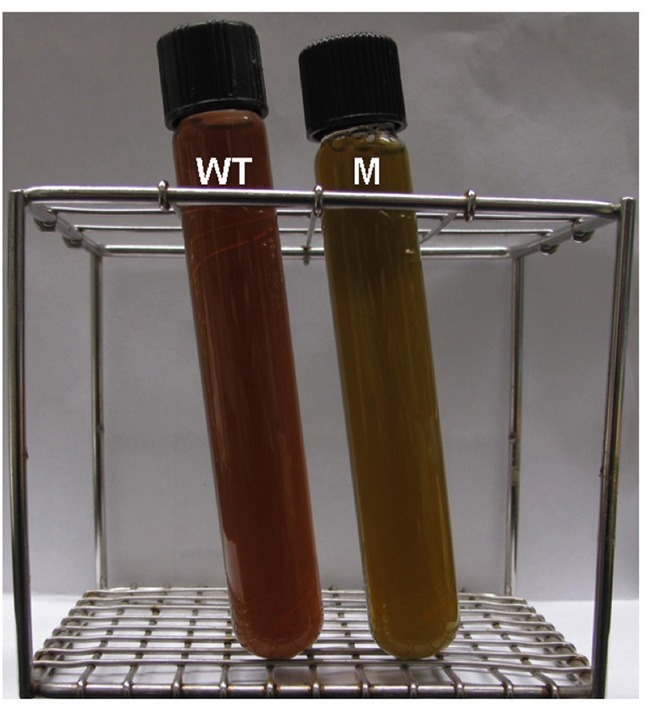
**Cultures of *C. limnaeum* 1677^T^ WT (tube WT) and the *bchU* mutant (tube M)**.

**Figure 4:**
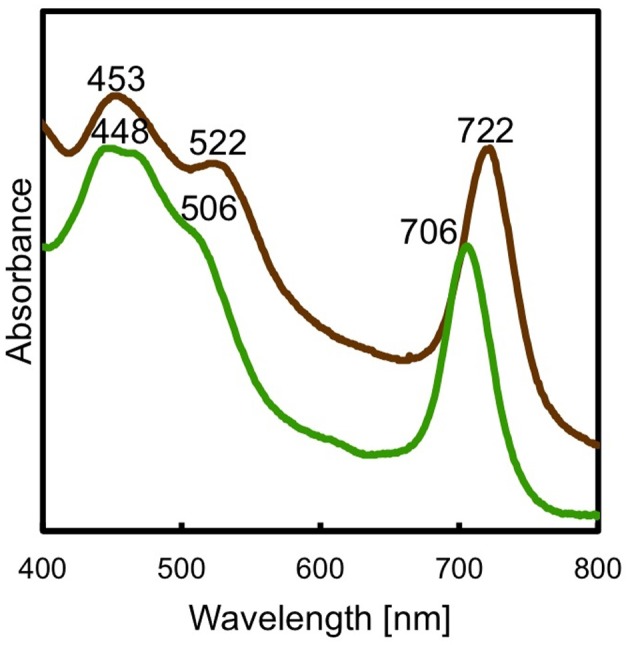
**Whole-cell absorption spectra of *C. limnaeum* 1677^T^ WT (brown line) and the *bchU* mutant (green line)**.

As shown in Figure 3, the resulting *bchU* mutant strain was greenish brown in color and was easily distinguishable from the WT, which was reddish brown in color. Figure 4 shows a comparison of the whole-cell absorption spectra for the WT and the *bchU* mutant. The WT cells had absorption maxima at 453, 522, and 722 nm, while the *bchU* mutant had absorption maxima at 448, 506, and 706 nm. As expected for the loss of the C-20 methyl group, the Q_y_ absorption band of the BChl aggregates in the chlorosomes was blue-shifted by ~16 nm.

**Figure 5:**
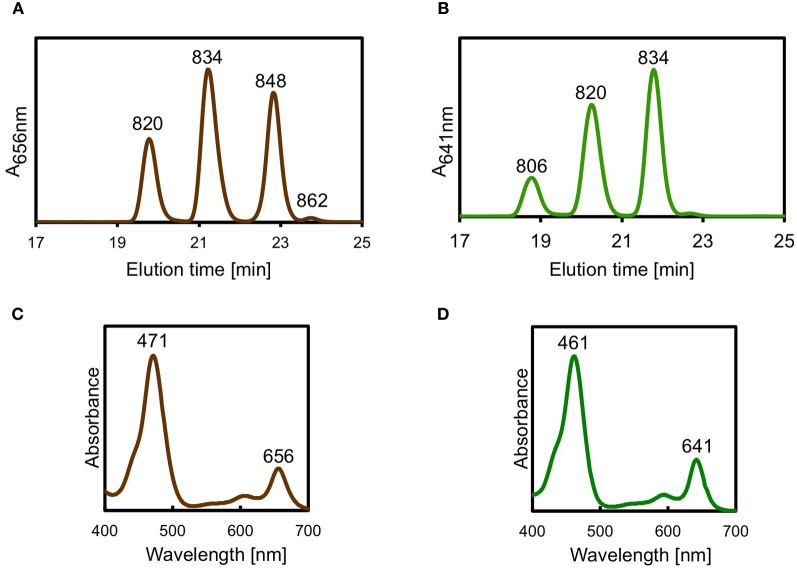
**Portion of HPLC elution profile for pigments extracted from *C. limnaeum* WT cells (panel **A**; monitored at 656 nm) and for *bchU* mutant (panel **B**; monitored at 641 nm).** The four peaks in panel **A** had identical absorption spectra (panel **C**); the *m/z* ratio of the BChls in each peak are indicated above and show that these are methyl homologs of BChl *e*_F_ (for additional details, see text). The four peaks in panel **B** also had identical absorption spectra (panel **D**); the *m/z* ratio of the BChls in three peaks are indicated [it was not possible to positively identify the 4th peak, which probably was [8-neo, 12-et]-BChl *f*_F_ (for additional details, see text)].

Figure 5A shows a portion of the elution profile for pigments extracted from WT *C. limnaeum* cells. Four BChl peaks were observed with elution times between 20 and 25 min, and all four had the same absorption spectra, with maxima at 471 and 656 nm (Figure 5C), which showed that these peaks are methylation homologs of BChl *e*. Mass spectroscopic analyses showed that these peaks corresponded to BChl *e* esterified with farnesol. Furthermore, it could be concluded from the mass spectroscopic data that all of the homologs were methylated at the C-12^1^ position. Thus, the four peaks, which had *m/z* values of 820, 834, 848, and 862, corresponded to [8-Et, 12-Et]-BChl *e*_F_, [8-Pr, 12-Et]-BChl *e*_F_, [8-Iso, 12-Et]-BChl *e*_F_, and [8-neo, 12-Et]-BChl *e*_F_. Figure 5B shows a portion of the elution profile of pigments extracted from cells of the *bchU* mutant. Although the relative proportions of the homologs were slightly different from those of the WT, four peaks were again observed. However, the elution times were shifted about 1 min earlier for each, which indicates that these compounds were less hydrophobic than BChl *e*_F_. The absorption spectra of these four peaks were identical and had absorption maxima at 461 and 641 nm, which are the expected values for BChl *f* (Figure 5D). Mass spectroscopic analyses confirmed this identification and showed that the *m/z* values for these homologs were 14 mass units smaller than the corresponding peaks for the WT, which is consistent with the loss of one methyl group (Note: Because of the low yield, it was not possible to determine the mass of the compound eluting at 22.5 min). The combination of these results indicated that these four peaks correspond to [8-Et, 12-Et]-BChl *f*_F_, [8-Pr, 12-Et]-BChl *f*_F_, [8-Iso, 12-Et]-BChl *f*_F_, and probably [8-Neo, 12-Et]-BChl *f*_F_.

### Isolation and characterization of chlorosomes from the *bchU* mutant

**Figure 6:**
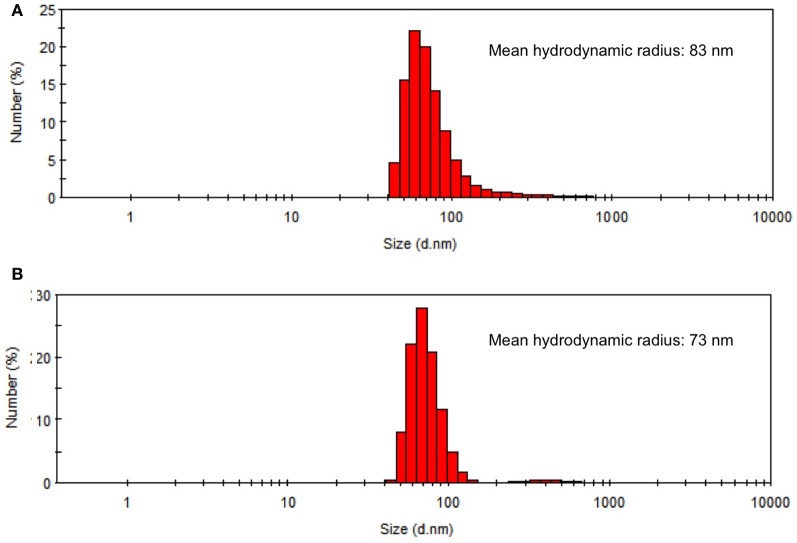
**Hydrodynamic diameter distribution of chlorosomes isolated from *C. limnaeum* WT cells (panel A) and the *bchU* mutant (panel B) as determined by dynamic light scattering.** The chlorosomes had similar mean hydrodynamic diameters but those from the WT were slightly larger (*d*_H_ = 83 nm) than those from the *bchU* mutant (*d*_H_ = 73 nm).

**Figure 7:**
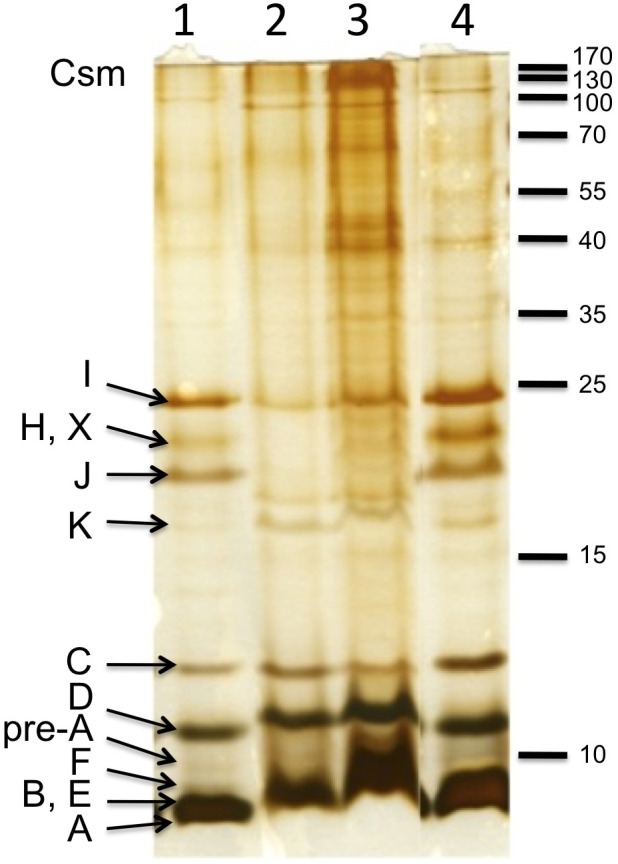
**SDS-PAGE analysis of polypeptide composition of chlorosomes from *C. tepidum* (lanes 1 and 4), *C. limnaeum bchU* mutant (lane 2), and *C. limnaeum* WT (lane 3).** The sizes of marker polypeptides are shown at the right in kDa. The identities of chlorosome proteins in *C. tepidum* chlorosomes are indicated at the left. Proteins corresponding to ~20 μg of BChl were loaded on each lane.

Chlorosomes were isolated from the WT and *bchU* mutant cells, and their properties were compared. As shown in Figure 6, chlorosomes from the WT and the *bchU* mutant had similar average hydrodynamic diameters (mean diameters, 83 and 73 nm, respectively) as estimated by light scattering. The polypeptide complement of chlorosomes from WT *C. limnaeum* differs from that of *C. tepidum* (Figure 7), but the polypeptide compositions of chlorosomes from the WT and *bchU* mutant strains were essentially indistinguishable (compare Figure 7, lanes 2 and 3). Thus, inactivation of the *bchU* gene and replacement of BChl *e*_F_ by BChl *f*_F_ did not alter the polypeptide composition of the chlorosome envelope in the *bchU* mutant.

**Figure 8:**
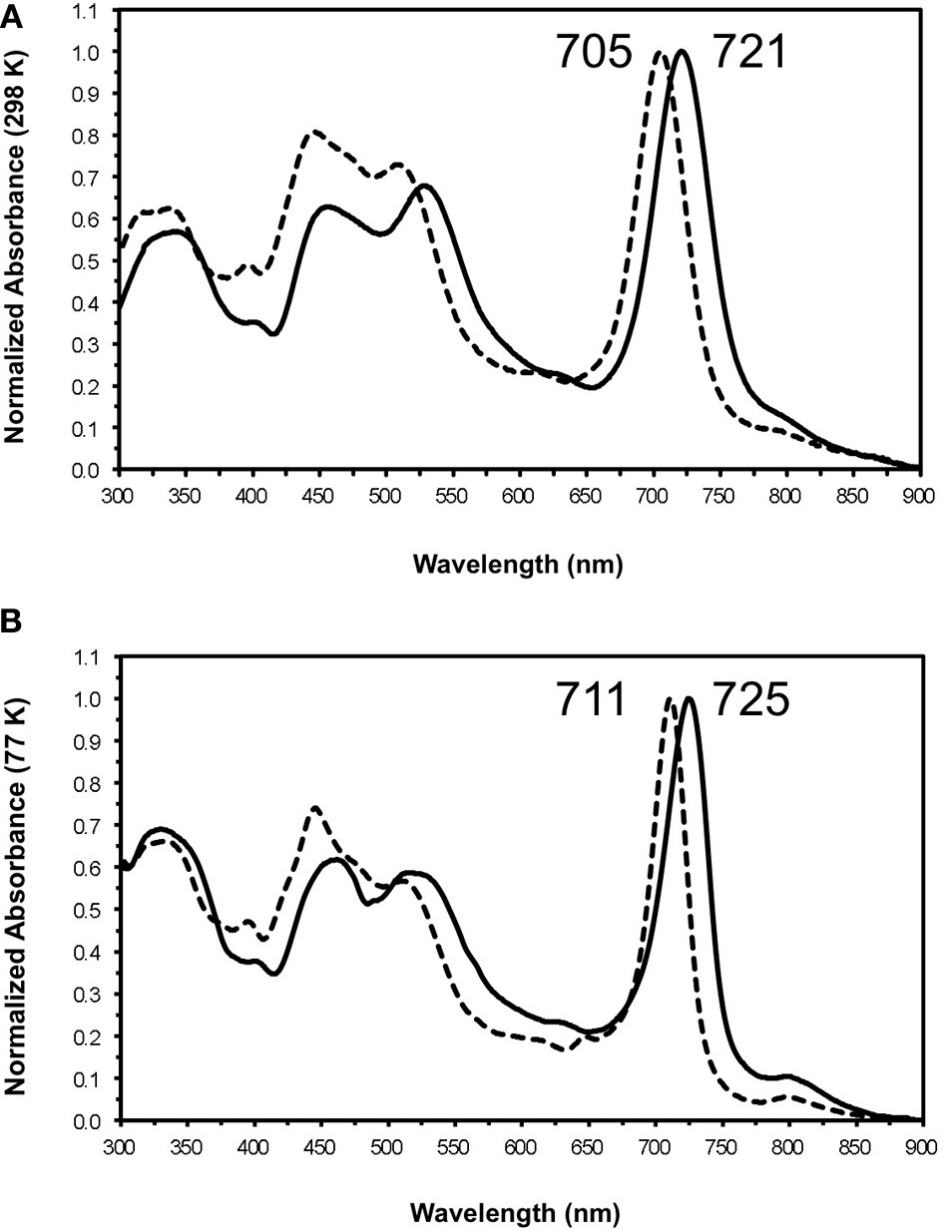
**Absorption spectra of chlorosomes from *C. limnaeum* WT (solid line) and *bchU* mutant (dotted line) at room temperature (A) and 77 K (B)**.

Figure 8 shows a comparison of the room temperature (Figure 8A) and low temperature (Figure 8B) absorption spectra for chlorosomes from the WT and the *bchU* mutant. Except for the baseplate absorption arising from BChl *a* at ~795 nm, the absorption maxima for chlorosomes from the *bchU* mutant are shifted to the blue. The 77 K absorption spectra provide sufficient peak resolution to show that the absorption of the BChl *a*-containing baseplate is unchanged between the WT and mutant. This indicates that the BChl *e* and BChl *f* oligomers transfer energy to the same type of acceptor in the chlorosome. Otherwise, the spectra of the mutant are very similar in shape to those for WT chlorosomes. WT chlorosomes had absorption maxima at 467, 528, 721, and 795 nm at room temperature (Figure 8A), while chlorosomes from the *bchU* mutant had absorption maxima at 446, 508, 705, and 794 nm. Similar absorption maxima were observed for the two samples at low temperature (Figure 8B). The half-band width of the Q_y_ absorption band was very slightly narrower for the chlorosomes from the *bchU* mutant (51 nm) than for those from the WT (55 nm).

**Figure 9:**
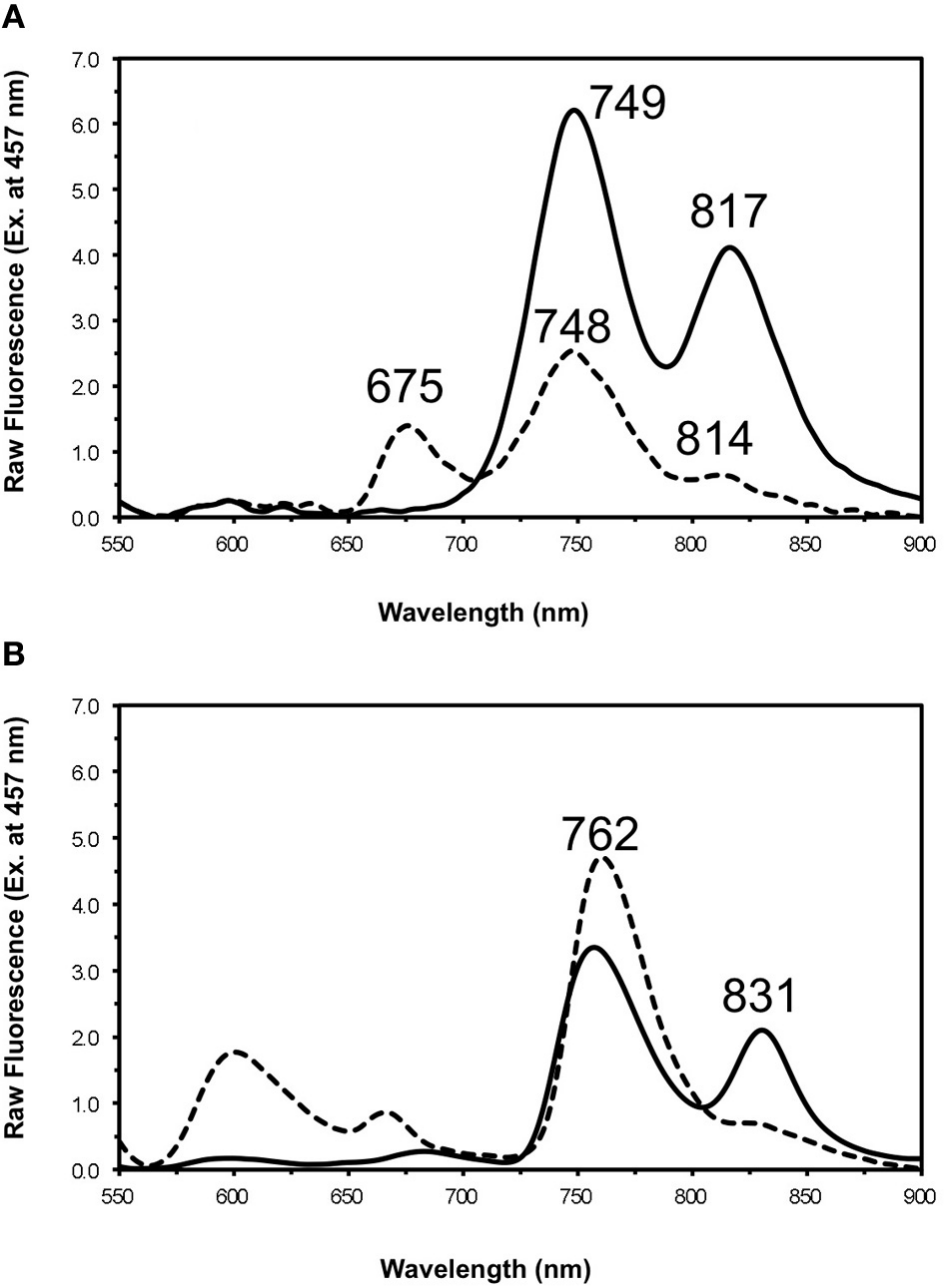
**Fluorescence emission spectra for chlorosomes from *C. limnaeum* WT at room temperature (panel **A**) and 77 K (panel B).** The excitation wavelength was 457 nm for each. Spectra of oxidized chlorosomes (dotted lines) were recorded in air-saturated buffer. The spectra of reduced chlorosomes (solid lines) were recorded after treatment with 25 mM sodium dithionite and incubation in the dark for 1 h.

**Figure 10:**
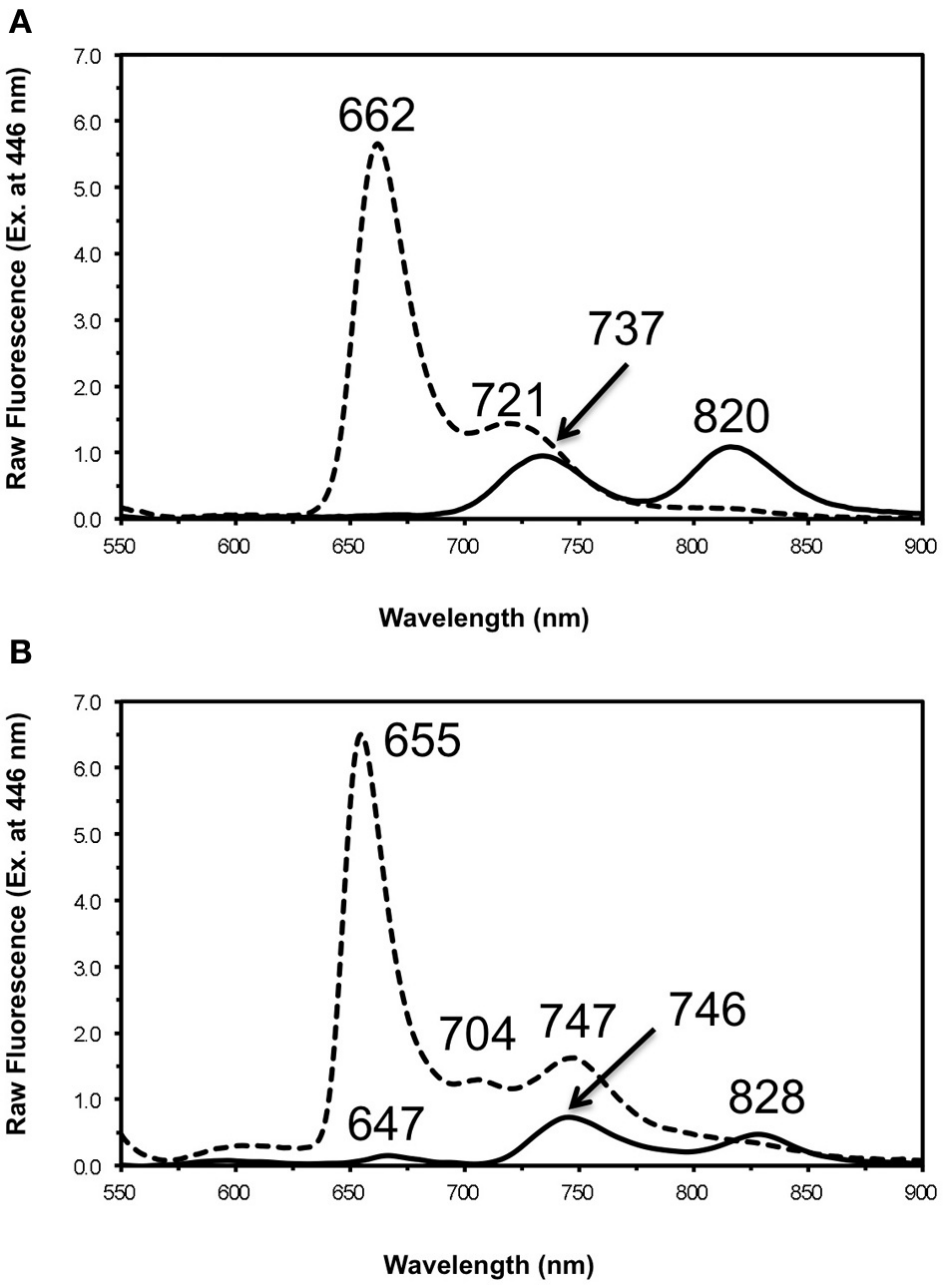
**Fluorescence emission spectra for chlorosomes from *C. limnaeum bchU* mutant at room temperature (panel A) and 77 K (panel B).** The excitation wavelength was 446 nm for each. Spectra of oxidized chlorosomes (dotted lines) were recorded in air-saturated buffer. The spectra of reduced chlorosomes (solid lines) were recorded after treatment with 25 mM sodium dithionite and incubation in the dark for 1 h.

The fluorescence emission spectra for chlorosomes isolated from the WT and the *bchU* mutant of *C. limnaeum*, under oxidizing and reducing conditions, are presented in Figures 9 and 10, respectively. Two emission peaks, with maxima at about 749 and 817 nm, are observed for reduced chlorosomes from the WT, and similar to *C. tepidum*, energy transfer from the BChl *e*_F_ aggregates to the BChl *a* associated with the baseplate was severely attenuated under oxidizing conditions (Figure 9A). For reduced chlorosomes from the *bchU* mutant, two emission peaks were also observed at room temperature, with maxima at about 737 and 820 nm (Figure 10A). Surprisingly, under oxidizing conditions, most of the fluorescence emission occurred at ~662 nm, which presumably arises from BChl *f* monomers, and a weaker secondary emission occurred at about 721 nm, but very little if any energy transfer to the BChl *a* of the baseplate occurred (Figure 10A). This behavior is different from that observed in chlorosomes of other GSB, which typically do not exhibit any significant fluorescence from pigment monomers. Figure 10B shows the fluorescence emission spectra of oxidized and reduced chlorosomes for the *bchU* mutant at 77 K. In addition to the emission from pigment monomers at 655 nm, the main chlorosome fluorescence band was resolved into two components that were centered at 704 and 747 nm. The 747 nm component is consistent with fluorescence emission from BChl *f* oligomers, and the 704 nm component is consistent with emission from a low energy vibrational state of monomeric BChl *f* (Figure 10B).

### Growth rate determinations for the WT and *bchU* strains of *C. limnaeum*

**Figure 11:**
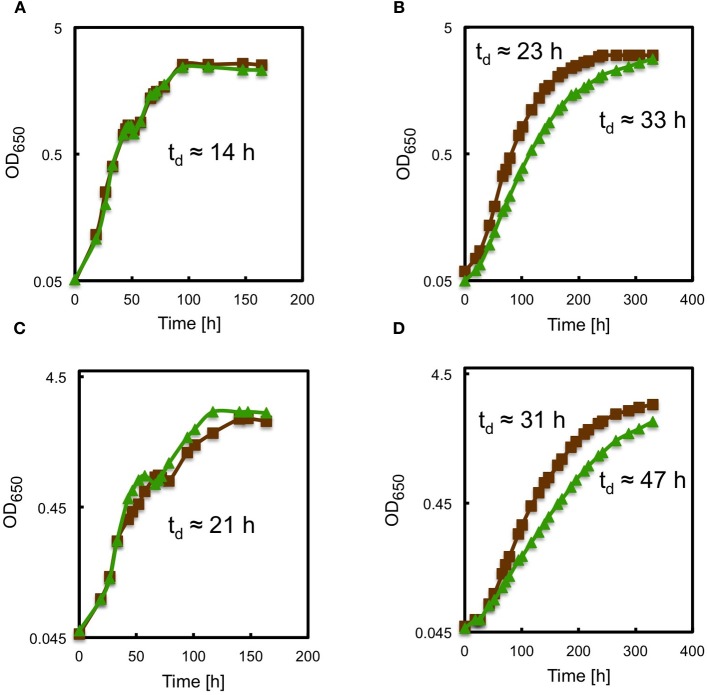
**Growth curves monitoring (OD_650 nm_) showing growth of *C. limnaeum* WT (brown lines) and *bchU* mutant (green lines) at irradiance values of 100 μmol photons m^−2^ s^−1^ (panels A and C) and 10 μmol photons m^−2^ s^−1^ (panels B and D).** For panels **A** and **B**, a tungsten lamp was used, and for panels **C** and **D**, cool white fluorescent lamps were used. For additional details, see text.

Because photoautotrophs depend directly on efficient light harvesting for growth, the growth rates of the WT and *bchU* mutant of *C. limnaeum* were measured to compare the light-harvesting properties of chlorosomes containing BChl *e*_F_ and BChl *f*_F_. As shown in Figure 11, when the WT and *bchU* mutant were grown at high irradiance (100 μmol photons m^−2^ s^−1^) with either tungsten light (Figures 11A,B) or cool white fluorescent light (Figures 11C,D), the two strains had virtually identical growth rates. Both strains grew faster with light provided by a tungsten source than with light from a cool white fluorescent source (~14-h doubling times *vs*. ~21-h doubling times). Each strain obviously grew much more slowly at low light intensity (10 μmol photons m^−2^ s^−1^), but at low light intensity the WT type strain grew about 40% faster than the *bchU* mutant, and this difference was independent of the type of light source used (Figures 11B,D). These results are consistent with the results discussed above, which showed that poorer energy transfer to the chlorosome baseplate and much poorer overall energy transfer efficiency occurs in chlorosomes of the *bchU* mutant.

## Discussion

Brown-colored GSB, those that assemble chlorosomes containing BChl *e* and usually isorenieratene as principal pigments, are typically found at extremely low irradiance values, which exclude the growth of most if not all other phototrophs, in anoxic layers with low redox potential (Vila and Abella, 1994; van Gemerden and Mas, 1995). In stratified freshwater lakes, these organisms typically occur at and/or below the chemocline (Vila and Abella, 2001; Tonolla et al., 2003), and in some exceptional cases, as in the Black Sea (Manske et al., 2005; Marschall et al., 2010), the photon fluxes where these organisms grow and persist can approach the extreme limit of one to ten photons absorbed per BChl molecule per day. That any organism is capable of growth under such extremely energy-limited conditions implicitly means that it must have a remarkably efficient light-harvesting system.

Progress in studying the light-harvesting apparatus of brown-colored GSB, however, has been severely limited because of the absence of a tractable genetic system for any of these organisms. Over a period of ~10 years, members of the Bryant laboratory systematically searched for an organism that provided the requisite properties required for the development of a genetic system: a reasonable growth rate on solid medium, a high plating efficiency, appropriate sensitivity to antibiotics, and the ability to take up DNA by natural transformation, electroporation, or conjugation and to recombine this DNA with chromosomal sequences. Several strains were tested and discarded over the years because none had the right combination of properties and none proved to be reliably transformable. Because of its phylogenetic relationship to *C. tepidum*, and because of its ability to use thiosulfate as an electron donor, *C. limnaeum* strain DSM 1677^T^ was eventually selected and tested for the development of a genetic system. As shown by this study, it is possible to produce transconjugants with suicide plasmids introduced by conjugative transfer from *E. coli* into this strain. We will publish the genome sequence of this organism elsewhere (Vogl et al., in preparation).

In *C. tepidum*, BchU is thought to catalyze the penultimate step in BChl *c* synthesis. The methylation reaction BchU catalyzes probably takes place immediately prior to the addition of the farnesyl tail to bacteriochlorophyllide *d* (Wada et al., 2006; Liu and Bryant, 2012). Because the enzymes that convert the 7-methyl group to the 7-formyl group of BChl *e* have not yet been identified, the order of the reactions leading from chlorophyllide *a* to bacteriochlorophyllide *e* are not yet known. Only BChl *f* was detected in the *bchU* mutant of *C. limnaeum*, and no other intermediates in the synthesis of BChl *e* were detected. Further studies will be required to elucidate the complete biosynthetic pathway for BChl *e*.

Inactivation of the *bchU* gene of *C. tepidum* produced several effects on chlorosomes (Maresca et al., 2004). Firstly, the absence of the C-20 methyl group caused a blue-shift of the Q_y_ absorption of the BChl *d* aggregates in chlorosomes. Secondly, the half-bandwidth of the Q_y_ absorption decreased, possibly because of a change in the supramolecular structure of the BChl aggregates in the chlorosomes (Ganapathy et al., 2009, 2012). Finally, the BChl of the cells of the *bchU* mutant was lower than that of wild-type cells grown at the same light intensity. The combination of these factors caused the mutant to grow significantly slower than the WT under all light conditions tested (Maresca et al., 2004). Some of these same effects may occur in the *bchU* mutant of *C. limnaeum*, although the effects of the mutation on the half-bandwidth and BChl content of the cells appear to be smaller than in *C. tepidum*.

At least thirteen major types of Chls and BChls are known to occur in bacteria: Chl *a*, Chl *b*, Chl *d*, Chl *f*, 8^1^-hydroxy-Chl *a*, 3,8-divinyl Chl *a*, 3,8-divinyl-Chl *b*; and BChl *a, b, c, d, e*, and *g* (Chew and Bryant, 2007; Liu and Bryant, 2012). Given the diversity of chemical structures and corresponding absorption properties represented by these molecules, it was a paradox why BChl *f*, which differs from BChl *e* only by the absence of a single methyl group at the C-20 methine carbon, had not been detected in any natural sample (Tamiaki et al., 2011). Although there is some overlap between the absorption of aggregated BChl *f* and Chl *a*, the absorption properties of chlorosomes containing BChl *f* do not overlap extensively with the absorption of other Chls except Chl *d* (Miyashita et al., 1996; Li et al., 2012) and Chl *f* (Chen et al., 2010; Li et al., 2012), which are rare Chls produced by only a few cyanobacteria. However, red light does not penetrate sufficiently deeply into most stratified water columns to reach the anoxic water layers where GSB can obtain the reduced sulfur compounds they require as electron donors for photoautotrophic growth (Vila and Abella, 1994, 2001). Finally, BChl *f* does not appear to have sufficiently unique absorption properties to define a natural light niche that would allow these organisms to outcompete other types of phototrophic bacteria (see Stomp et al., 2007). Thus, BChl *f* may not occur naturally because anoxic environments where sulfide concentrations are high, redox potentials are low, and appropriate irradiance characteristics occur to define a unique light niche are either uncommon or non-existent.

The results from this study suggest several other possible reasons why BChl *f* has not been found in nature. Firstly, the aggregated forms of BChl *f*_F_ in chlorosomes are only slightly red-shifted relative to Chl *a*, and because all oxygenic photosynthetic organisms produce Chl *a*, GSB living deep in the anoxic layers of stratified lakes and producing BChl *f*_F_ would receive light that would be strongly filtered by the Chl *a* associated with cyanobacteria and algae in the oxic layer of stratified systems. Moreover, carotenoids associated with light-harvesting proteins in eukaryotic algae and prokaryotes would additionally filter some blue light that might otherwise be absorbed by BChl *f*. Secondly, there is a very large energy gap, ~90 nm, between the BChl *f*_F_ aggregates in chlorosomes and the BChl *a* associated with CsmA in the baseplate. Although the transfer is downhill energy-wise, this gap may be too large to allow efficient energy transfer (this will be explicitly explored in future studies). This conclusion is supported by initial inspection of the fluorescence emission spectra for the fully reduced chlorosomes. Energy transfer appears to be less efficient in the chlorosomes of the *bchU* mutant than in those of the WT, because the amplitude of the emission from the BChl *a* associated with the CsmA baseplate is lower for the mutant than the WT at equal absorption. The molar extinction coefficient for BChls *e* and *f* are expected to be similar and should not significantly factor into this difference because of the high degree of structural similarity for the two pigments. Finally, similar to previous observations for other chlorosomes (Wang et al., 1990; Blankenship et al., 1993; Frigaard et al., 1997; Garcia Costas et al., 2011), energy transfer in both the WT and the *bchU* mutant was extremely sensitive to the oxidation state of the chlorosomes. The presence of even low amounts of oxygen could be sufficient to cause significant quenching of energy transfer in these organisms. Whatever the actual cause(s), the growth rate studies presented here demonstrate convincingly that cells synthesizing BChl *f* could not compete effectively with WT cells producing BChl *e* for their chlorosomes in any natural, light-limited environment. Furthermore, any mutant that did arise by inactivation of the *bchU* gene would quickly be eliminated from the natural community, because those cells would be unable to compete effectively with cells producing BChl *e* for the light energy required for growth.

## Conclusion

We report here the construction of a *bchU* mutant in *C. limnaeum*, the first targeted mutation constructed in a brown-colored GSB. This mutant produced chlorosomes containing aggregates of BChl *f*_F_, a pigment that had not previously been reported to occur in any natural system. The *bchU* mutant grew much slower than the WT at low irradiance values. Energy transfer from the BChl *f*_F_ aggregates to the BChl *a* in the chlorosome baseplates was less efficient than in chlorosomes containing BChl *e*_F_ aggregates. It appears that energy transfer is less efficient in the chlorosomes of the *bchU* mutant, but the causes of that inefficiency are currently under investigation and will be the subject of further studies. Ongoing static and time-resolved spectroscopic studies will hopefully provide a more complete explanation for the poor light-harvesting properties of BChl *f*. Whatever the reason(s), it is obvious that bacteria producing this pigment could not compete well with BChl *e*-producing strains in natural light-limited environments and would be quickly eliminated from any natural population in which they arose because BChl *f* does not allow the cells to occupy a unique light niche.

### Conflict of interest statement

The authors declare that the research was conducted in the absence of any commercial or financial relationships that could be construed as a potential conflict of interest.

## Abbreviations

BChl: bacteriochlorophyll
(B)Chl: bacteriochlorophyll or chlorophyll
Chl: chlorophyll
Et: ethyl
F: farnesyl
GSB: green sulfur bacteria
Iso: isobutyl
Neo: neopentyl
OD: optical density at the specified wavelength
PAGE: polyacrylamide gel electrophoresis
PCR: polymerase chain reaction
Pr: propionyl
SDS: sodium dodecylsulfate.

## References

[B1] Airs R. L. Keely B. J. (2000). A novel approach for sensitivity enhancement in atmospheric pressure chemical ionisation liquid chromatography/mass spectrometry of chlorophylls. Rapid Commun. Mass Spectrom. 14, 125–128 10.1002/(SICI)1097-0231(20000215)14:3<125::AID-RCM847>3.0.CO;2-6 10637416

[B2] Azai C. Tsukatani Y. Harada J. Oh-oka H. (2009). Sulfur oxidation in mutants of the photosynthetic green sulfur bacterium *Chlorobium tepidum* devoid of cytochrome *c*-554 and SoxB. Photosynth. Res. 100, 57–65 10.1007/s11120-009-9426-2 19421892

[B3] Beatty J. T. Overmann J. Lince M. T. Manske A. K. Lang A. S. Blankenship R. E. Van Dover C. L. Martinson T. A. Plumley F. G. (2005). An obligately photosynthetic bacterial anaerobe from a deep-sea hydrothermal vent. Proc. Natl. Acad. Sci. U.S.A. 102, 9306–9310 10.1073/pnas.0503674102 15967984 PMC1166624

[B4] Blankenship R. E. (2004). Identification of a key step in the biosynthetic pathway of bacteriochlorophyll *c* and its implications for other known and unknown green sulfur bacteria. J. Bacteriol. 186, 5187–5188 10.1128/JB.186.16.5187-5188.2004 15292118 PMC490940

[B5] Blankenship R. E. Cheng P. Causgrove T. P. Brune D. C. Wang S. H. H. Choh J.-U. Wang J. (1993). Redox regulation of energy transfer efficiency in antennas of green photosynthetic bacteria. Photochem. Photobiol. 57, 103–107 10.1111/j.1751-1097.1993.tb02263.x 11537865

[B6] Blankenship R. E. Matsuura K. (2003). Antenna complexes from green photosynthetic bacteria, in Advances in Photosynthesis and Respiration, Vol. 13, Light-Harvesting Antennas, eds Green B. R. Parson W. W. (Dordrecht: Kluwer), 195–217 10.1023/B:PRES.0000004329.26878.d8

[B7] Blum H. Beier H. Gross H. J. (1987). Improved silver staining of plant proteins, RNA and DNA in polyacrylamide gels. Electrophoresis 8, 93–99

[B8] Bryant D. A. Garcia Costas A. M. Maresca J. A. Gomez Maqueo Chew A. G. Klatt C. G. Bateson M. M. Tallon L. J. Hostetler J. Nelson W. C. Heidelberg J. F. Ward D. M. (2007). *Candidatus Chloracidobacterium thermophilum*: an aerobic phototrophic *Acidobacterium*. Science 317, 523–526 10.1126/science.1143236 17656724

[B9] Bryant D. A. Liu Z. Li T. Zhao F. Garcia Costas A. M. Klatt C. G. Ward D. M. Frigaard N.-U. Overmann J. (2012). Comparative and functional genomics of anoxygenic green bacteria from the taxa Chlorobi, Chloroflexi, and Acidobacteria, in Advances in Photosynthesis and Respiration, Vol. 35, Functional Genomics and Evolution of Photosynthetic Systems, eds Burnap R. L. Vermaas W. (Dordrecht: Springer), 47–102

[B10] Chan L. K. Morgan-Kiss R. M. Hanson T. E. (2009). Functional analysis of three sulfide: quinone oxidoreductase homologs in *Chlorobaculum tepidum*. J. Bacteriol. 191, 1026–1034 10.1128/JB.01154-08 19028893 PMC2632091

[B11] Chan L. K. Weber T. S. Morgan-Kiss R. M. Hanson T. E. (2008). A genomic region required for phototrophic thiosulfate oxidation in the green sulfur bacterium *Chlorobium tepidum* (syn. *Chlorobaculum tepidum*). Microbiology 154, 818–829 10.1099/mic.0.2007/012583-0 18310028

[B12] Chen M. Schliep M. Willows R. D. Cai Z. L. Neilan B. A. Scheer H. (2010). A red-shifted chlorophyll. Science 329, 1318–1319 10.1126/science.1191127 20724585

[B22] Chew A. G. Bryant D. A. (2007). Chlorophyll biosynthesis in bacteria: the origins of structural and functional diversity. Annu. Rev. Microbiol. 61, 113–129 10.1146/annurev.micro.61.080706.093242 17506685

[B13] Eisen J. A. Nelson K. E. Paulsen I. T. Heidelberg J. F. Wu M. Dodson R. J. Deboy R. Gwinn M. L. Nelson W. C. Haft D. H. Hickey E. K. Peterson J. D. Durkin A. S. Kolonay J. L. Yang F. Holt I. Umayam L. A. Mason T. Brenner M. Shea T. P. Parksey D. Feldblyum T. V. Hansen C. L. Craven M. B. Radune D. Khouri H. Fujii C. Y. White O. Venter J. C. Volfovsky N. Gruber T. M. Ketchum K. A. Tettelin H. Bryant D. A. Fraser C. M. (2002). The complete genome sequence of the green sulfur bacterium *Chlorobium tepidum*. Proc. Natl. Acad. Sci. U.S.A. 99, 9509–9514 10.1073/pnas.132181499 12093901 PMC123171

[B14] Frigaard N.-U. Bryant D. A. (2001). Chromosomal gene inactivation in the green sulfur bacterium *Chlorobium tepidum* by natural transformation. Appl. Environ. Microbiol. 67, 2538–2544 10.1128/AEM.67.6.2538-2544.2001 11375161 PMC92905

[B15] Frigaard N.-U. Bryant D. A. (2006). Chlorosomes: antenna organelles in green photosynthetic bacteria, in Microbiology Monographs, Vol. 2, Complex Intracellular Structures in Prokaryotes, ed Shively J. (Berlin: Springer), 79–114

[B16] Frigaard N.-U. Li H. Milks K. J. Bryant D. A. (2004a). Nine mutants of *Chlorobium tepidum* each unable to synthesize a different chlorosome protein still assemble functional chlorosomes. J. Bacteriol. 186, 646–653 10.1128/JB.186.3.646-653.2004 14729689 PMC321489

[B17] Frigaard N.-U. Maresca J. A. Yunker C. E. Jones A. D. Bryant D. A. (2004b). Genetic manipulation of carotenoid biosynthesis in the green sulfur bacterium *Chlorobium tepidum*. J. Bacteriol. 186, 5210–5220 10.1128/JB.186.16.5210-5220.2004 15292122 PMC490927

[B18] Frigaard N.-U. Takaichi S. Hirota M. Shimada K. Matsuura K. (1997). Quinones in chlorosomes of green sulfur bacteria and their role in the redox-dependent fluorescence studied in chlorosome-like bacteriochlorophyll *c* aggregates. Arch. Microbiol. 167, 343–349

[B19] Ganapathy S. Oostergetel G. T. Wawrzyniak P. K. Reus M. Gomez Maqueo Chew A. Buda F. Boekema E. J. Bryant D. A. Holzwarth A. R. de Groot H. J. M. (2009). Alternating *syn-anti* bacteriochlorophylls form concentric helical nanotubes in chlorosomes. Proc. Natl. Acad. Sci. U.S.A. 106, 8525–8530 10.1073/pnas.0903534106 19435848 PMC2680731

[B20] Ganapathy S. Reus M. Oostergetel G. Wawrzyniak P. K. Tsukatani Y. Gomez Maqueo Chew A. Buda F. Bryant D. A. Holzwarth A. R. de Groot H. J. M. (2012). Self-assembly of BChl *c* in chlorosomes of the green sulfur bacterium, *Chlorobaculum tepidum*: a comparison of the *bchQR* mutant and the wild type. Biochemistry 51, 4488–4498 10.1021/bi201817x 22577986

[B21] Garcia Costas A. M. Tsukatani Y. Romberger S. P. Oostergetel G. Boekema E. Golbeck J. H. Bryant D. A. (2011). Ultrastructural analysis and identification of envelope proteins of “*Candidatus Chloracidobacterium thermophilum*” chlorosomes. J. Bacteriol. 193, 6701–6711 10.1128/JB.06124-11 21965575 PMC3232888

[B23] Gomez Maqueo Chew A. Frigaard N.-U. Bryant D. A. (2007). Bacteriochlorophyllide *c* C-8^2^ and C-12^1^ methyltransferases are essential for adaptation to low light in *Chlorobaculum tepidum*. J. Bacteriol. 189, 6176–6184 10.1128/JB.00519-07 17586634 PMC1951906

[B24] Gregersen L. H. Bryant D. A. Frigaard N.-U. (2011). Components and evolution of oxidative sulfur metabolism in green sulfur bacteria. Front. Microbio. 2:116 10.3389/fmicb.2011.00116 21833341 PMC3153061

[B25] Holkenbrink C. Barbas S. O. Mellerup A. Otaki H. Frigaard N.-U. (2011). Sulfur globule oxidation in green sulfur bacteria is dependent on the dissimilatory sulfite reductase system. Microbiology 157, 1229–1239 10.1099/mic.0.044669-0 21233162

[B26] Imhoff J. F. (2003). Phylogenetic taxonomy of the family *Chlorobiaceae* on the basis of 16S rRNA and *fmo* (Fenna-Matthews-Olson protein) gene sequences. Int. J. Syst. Evol. Microbiol. 53, 941–951 10.1099/ijs.0.02403-0 12892110

[B27] Lenz O. Schwartz E. Dernedde J. Eitinger M. Friedrich B. (1994). The *Alcaligenes eutrophus* H16 *hoxX* gene participates in hydrogenase regulation. J. Bacteriol. 176, 4385–4393 8021224 10.1128/jb.176.14.4385-4393.1994 PMC205652

[B28] Li H. Bryant D. A. (2009). Envelope proteins of the CsmB/CsmF and CsmC/CsmD motif families help determine the size, shape and composition of chlorosomes in *Chlorobaculum tepidum*. J. Bacteriol. 191, 7109–7120 10.1128/JB.00707-09 19749040 PMC2772486

[B29] Li H. Jubelirer S. Garcia Costas A. M. Frigaard N.-U. Bryant D. A. (2009). Multiple antioxidant proteins protect *Chlorobaculum tepidum* against oxygen and reactive oxygen species. Arch. Microbiol. 191, 853–867 10.1007/s00203-009-0514-7 19784828

[B30] Li Y. Scales N. Blankenship R. E. Willows R. D. Chen M. (2012). Extinction coefficient for red-shifted chlorophylls: chlorophyll *d* and chlorophyll *f*. Biochim. Biophys. Acta 1817, 1292–1298 10.1016/j.bbabio.2012.02.026 22395150

[B31] Liu Z. Bryant D. A. (2012). Biosynthesis and assembly of bacteriochlorophyll c in green bacteria: theme and variations, in Handbook of Porphyrin Science, Vol. 20, eds Kadish K. M. Smith K. M. Guilard R. (Hackensack, NJ: World Scientific Publishing), 108–142

[B32] Manske A. K. Glaeser J. Kuypers M. M. Overmann J. (2005). Physiology and phylogeny of green sulfur bacteria forming a monospecific phototrophic assemblage at a depth of 100 meters in the Black Sea. Appl. Environ. Microbiol. 71, 8049–8060 10.1128/AEM.71.12.8049-8060.2005 16332785 PMC1317439

[B33] Maresca J. A. Gomez Maqueo Chew A. Ros Ponsatí M. Frigaard N.-U. Ormerod J. G. Jones A. D. Bryant D. A. (2004). The *bchU* gene of *Chlorobium tepidum* encodes the bacteriochlorophyll C-20 methyltransferase. J. Bacteriol. 186, 2558–2566 10.1128/JB.186.9.2558-2566.2004 15090495 PMC387796

[B34] Maresca J. A. Graham J. E. Bryant D. A. (2008). Carotenoid biosynthesis in chlorophototrophs: the biochemical and genetic basis for structural diversity. Photosynth. Res. 97, 121–140 10.1007/s11120-008-9312-3 18535920

[B35] Marschall E. Jogler M. Hessge U. Overmann J. (2010). Large-scale distribution and activity patterns of an extremely low-light adapted population of green sulfur bacteria in the Black Sea. Environ. Microbiol. 12, 1348–1362 10.1111/j.1462-2920.2010.02178.x 20236170

[B36] Martinez-Planells A. Arellano J. B. Borrego C. M. López-Iglesias C. Gich F. Garcia-Gil J. (2002). Determination of the topography and biometry of chlorosomes by atomic force microscopy. Photosynth. Res. 71, 83–90 10.1023/A:1014955614757 16228503

[B37] Miyashita H. Ikemoto H. Kurano N. Adachi K. Chihara M. Miyachi S. (1996). Chlorophyll *d* as a major pigment. Nature 383, 402

[B38] Montaño G. A. Bowen B. P. LaBelle J. T. Woodbury N. W. Pizziconi V. B. Blankenship R. E. (2003). Characterization of *Chlorobium tepidum* chlorosomes: a calculation of bacteriochlorophyll *c* per chlorosome and oligomer modeling. Biophys. J. 85, 2560–2565 10.1016/S0006-3495(03)74678-5 14507718 PMC1303479

[B39] Oostergetel G. T. van Amerongen H. Boekema E. J. (2010). The chlorosome: a prototype for efficient light harvesting in photosynthesis. Photosynth. Res. 104, 245–255 10.1007/s11120-010-9533-0 20130996 PMC2882566

[B40] Overmann J. Pfennig N. (1989). *Pelodictyon phaeoclathratiforme* sp. nov., a new brown colored member of the *Chlorobiaceae* forming net-like colonies. Arch. Microbiol. 152, 401–406

[B41] Simon R. Priefer U. Pühler A. (1983). A broad host range mobilization system for *in vivo* genetic engineering: transposon mutagenesis in gram-negative bacteria. Nat. Biotechnol. 1, 784–791

[B42] Schägger H. von Jagow G. (1987). Tricine-sodium dodecylsulfate-polyacrylamide gel electrophoresis for the separation of proteins in the range from 1 to 100 kDa. Anal. Biochem. 166, 369–379 2449095 10.1016/0003-2697(87)90587-2

[B43] Stomp M. Huisman J. Stal L. J. Matthijs H. C. (2007). Colorful niches of phototrophic microorganisms shaped by vibrations of the water molecule. ISME J. 1, 271–282 10.1038/ismej.2007.59 18043638

[B44] Tamiaki H. Komada J. Kunieda M. Fukai K. Yoshitomi T. Harada J. Mizoguchi T. (2011). *In vitro* synthesis and characterization of bacteriochlorophyll-*f* and its absence in bacteriochlorophyll-*e* producing organisms. Photosynth. Res. 107, 133–138 10.1007/s11120-010-9603-3 21161597

[B45] Tanaka A. Ito H. Tanaka R. Tanaka N. K. Yoshida K. Okada K. (1998). Chlorophyll *a* oxygenase (CAO) is involved in chlorophyll *b* formation from chlorophyll *a*. Proc. Natl. Acad. Sci. U.S.A. 95, 12719–12723 10.1073/pnas.95.21.12719 9770552 PMC22897

[B46] Tanaka R. Tanaka A. (2011). Chlorophyll cycle regulates the construction and destruction of the light-harvesting complexes. Biochim. Biophys. Acta 1807, 968–976 10.1016/j.bbabio.2011.01.002 21216224

[B47] Tonolla M. Peduzzi S. Hahn D. Peduzzi R. (2003). Spatio-temporal distribution of phototrophic sulfur bacteria in the chemocline of meromictic Lake Cadagno (Switzerland). FEMS Microbiol. Ecol. 43, 89–98 10.1111/j.1574-6941.2003.tb01048.x 19719699

[B48] Tsukatani Y. Miyamoto R. Itoh S. Oh-Oka H. (2004). Function of PscD subunit in a homodimeric reaction center complex of the photosynthetic green sulfur bacterium *Chlorobium tepidum* studied by insertional gene inactivation. Regulation of energy transfer and ferredoxin-mediated NADP^+^ reduction on the cytoplasmic side. J. Biol. Chem. 279, 51122–51130 15371432 10.1074/jbc.M410432200

[B49] van Gemerden H. Mas J. (1995). Ecology of phototrophic sulfur bacteria, in Advances in Photosynthesis and Respiration, Vol. 2, Anoxygenic Photosynthetic Bacteria, eds Blankenship R. E. Madigan M. T. Bauer C. E. (Dordrecht: Springer), 49–85

[B50] Vassilieva E. V. Stirewalt V. L. Jakobs C. U. Frigaard N.-U. Inoue-Sakamoto K. Baker M. A. Sotak A. Bryant D. A. (2002). Subcellular localization of chlorosome proteins in *Chlorobium tepidum* and characterization of three new chlorosome proteins: CsmF, CsmH, and CsmX. Biochemistry 41, 4358–4370 11914082 10.1021/bi012051u

[B51] Vila X. Abella C. A. (1994). Effects of light quality on the physiology and the ecology of planktonic green sulfur bacteria in lakes. Photosynth. Res. 41, 53–65 10.1007/BF02184145 24310013

[B52] Vila X. Abella C. A. (2001). Light-harvesting adaptations of planktonic phototroph micro-organisms to different light quality conditions. Hydrobiologia 452, 15–30

[B53] Wada K. Yamaguchi H. Harada J. Niimi K. Osumi S. Saga Y. Oh-Oka H. Tamiaki H. Fukuyama K. (2006). Crystal structures of BchU, a methyltransferase involved in bacteriochlorophyll *c* biosynthesis, and its comples with S-adenosylhomocysteine: implications for reaction mechanism. J. Mol. Biol. 360, 839–849 10.1016/j.jmb.2006.05.057 16797589

[B54] Wang J. Brune D. C. Blankenship R. E. (1990). Effects of oxidants and reductants on the efficiency of excitation transfer in green photosynthetic bacteria. Biochim. Biophys. Acta 1015, 457–463 11536463 10.1016/0005-2728(90)90079-j

[B55] Wahlund T. M. Madigan M. T. (1995). Genetic transfer by conjugation in the thermophilic green sulfur bacterium *Chlorobium tepidum*. J. Bacteriol. 177, 2583–2588 7730296 10.1128/jb.177.9.2583-2588.1995 PMC176923

